# Immediate and durable effects of maternal tobacco consumption alter placental DNA methylation in enhancer and imprinted gene-containing regions

**DOI:** 10.1186/s12916-020-01736-1

**Published:** 2020-10-07

**Authors:** Sophie Rousseaux, Emie Seyve, Florent Chuffart, Ekaterina Bourova-Flin, Meriem Benmerad, Marie-Aline Charles, Anne Forhan, Barbara Heude, Valérie Siroux, Remy Slama, Jorg Tost, Daniel Vaiman, Saadi Khochbin, Johanna Lepeule, the EDEN Mother-Child Cohort Study Group, I. Annesi-Maesano, J. Y. Bernard, J. Botton, M-A Charles, P. Dargent-Molina, B. de Lauzon-Guillain, P. Ducimetière, M. de Agostini, B. Foliguet, A. Forhan, X. Fritel, A. Germa, V. Goua, R. Hankard, B. Heude, M. Kaminski, B. Larroque, N. Lelong, J. Lepeule, G. Magnin, L. Marchand, C. Nabet, F. Pierre, R. Slama, M. J. Saurel-Cubizolles, M. Schweitzer, O. Thiebaugeorges

**Affiliations:** 1 Université Grenoble Alpes, Inserm, CNRS, IAB, 38000 Grenoble, France; 2 Université de Paris, Centre for Research in Epidemiology and Statistics (CRESS), INSERM, INRAE, Paris, France; 3 Laboratory for Epigenetics and Environment, Centre National de Recherche en Génomique Humaine, CEA – Institut de Biologie François Jacob, Evry, France; 4 Genomics, Epigenetics and Physiopathology of Reproduction, Institut Cochin, U1016 Inserm – UMR 8104 CNRS – Paris-Descartes University, Paris, France

**Keywords:** Placenta, DNA methylation, Pregnancy, Smoking, Epigenome-wide association study, Molecular epidemiology

## Abstract

**Background:**

Although exposure to cigarette smoking during pregnancy has been associated with alterations of DNA methylation in the cord blood or placental cells, whether such exposure before pregnancy could induce epigenetic alterations in the placenta of former smokers has never been investigated.

**Methods:**

Our approach combined the analysis of placenta epigenomic (ENCODE) data with newly generated DNA methylation data obtained from 568 pregnant women, the largest cohort to date, either actively smoking during their pregnancy or formerly exposed to tobacco smoking.

**Results:**

This strategy resulted in several major findings. First, among the 203 differentially methylated regions (DMRs) identified by the epigenome-wide association study, 152 showed “reversible” alterations of DNA methylation, only present in the placenta of current smokers, whereas 26 were also found altered in former smokers, whose placenta had not been exposed directly to cigarette smoking. Although the absolute methylation changes were smaller than those observed in other contexts, such as in some congenital diseases, the observed alterations were consistent within each DMR. This observation was further supported by a demethylation of *LINE-1* sequences in the placentas of both current (beta-coefficient (*β*) (95% confidence interval (CI)), − 0.004 (− 0.008; 0.001)) and former smokers (*β* (95% CI), − 0.006 (− 0.011; − 0.001)) compared to nonsmokers. Second, the 203 DMRs were enriched in epigenetic marks corresponding to enhancer regions, including monomethylation of lysine 4 and acetylation of lysine 27 of histone H3 (respectively H3K4me1 and H3K27ac). Third, smoking-associated DMRs were also found near and/or overlapping 10 imprinted genes containing regions (corresponding to 16 genes), notably including the *NNAT*, *SGCE/PEG10*, and *H19/MIR675* loci.

**Conclusions:**

Our results pointing towards genomic regions containing the imprinted genes as well as enhancers as preferential targets suggest mechanisms by which tobacco could directly impact the fetus and future child. The persistence of significant DNA methylation changes in the placenta of former smokers supports the hypothesis of an “epigenetic memory” of exposure to cigarette smoking before pregnancy. This observation not only is conceptually revolutionary, but these results also bring crucial information in terms of public health concerning potential long-term detrimental effects of smoking in women.

## Background

Despite increasing awareness of smoking-associated risks in pregnancy and although smoking cessation is recognized as one of the most effective actions for improving mothers’ and children’s health [1], between 5 and 20% of women continue to smoke during pregnancy in the USA and Europe [2, 3], with a prevalence of about 8% in Germany, 14% in Spain, 12% in the UK, and 17% in France [3]. Maternal smoking during pregnancy is the most frequent preventable cause of adverse pregnancy outcomes [4] including placental abruption, placenta previa [5], preterm delivery [6], and some congenital anomalies [7]. It has also been causally associated with intrauterine growth restriction [8]. In the long term, maternal smoking during pregnancy is associated with adverse outcomes on a child’s respiratory [9, 10] and cardiometabolic [11, 12] health, neurodevelopment [13], and cancer [14–16]. Despite this large amount of evidence supporting the effects of maternal smoking on the placenta, fetus, and child, the molecular mechanisms involved in these effects remain poorly understood.

Intrauterine life is a critical period of plasticity during which environmental insults can alter the developmental programming via epigenetic phenomena, with immediate effects visible at birth or delayed effects that appear in childhood, puberty, or adulthood [17]. The most explored epigenetic mark so far has been the methylation of DNA, a modification known to be involved in the control of gene expression. More specifically, DNA methylation involves the addition of a methyl group to a cytosine, which in mammals is located upstream guanine residues (a “CpG” dinucleotide) on the DNA molecule. Modifications of DNA methylation can be replicated through cell divisions and can persist even in the absence of the cause that established them (biological memory) [18]. Although DNA methylation is relatively stable in somatic cells, where it is transmitted through mitotic divisions, there are specific periods during development when the methylation pattern is reprogrammed, such as after fertilization in the pre-implantation embryo [19]. Maternal smoking during pregnancy has been associated with DNA methylation levels in buccal cells of child offspring [20], in the blood of adolescent offspring [21], and in peripheral blood granulocytes in adult women [22]. In neonates, most studies on tobacco smoking during pregnancy have focused on cord blood DNA methylation [23–26]. As a transient organ, the placenta is particularly interesting since it may provide a “molecular archive” of the prenatal environment [27], as recently shown for maternal smoking during pregnancy [28].

**Fig. 1:**
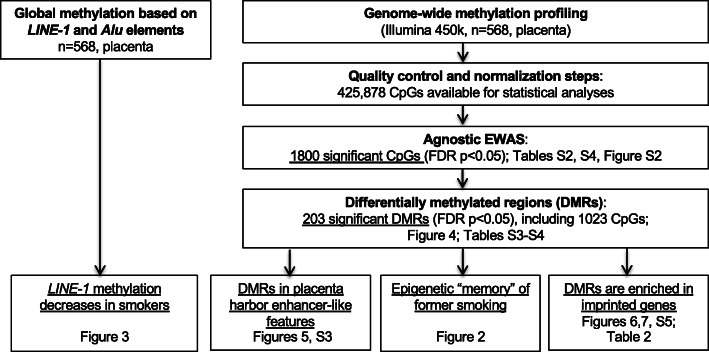
Workflow of the study

A few studies have investigated the association between placental DNA methylation and maternal smoking during pregnancy [29–33]. Most studies were restricted to the investigation of candidate CpGs or low-density genome-wide analyses, and many of them were conducted on a small sample size. Importantly, these studies only included women who smoked throughout their pregnancy or who quit smoking during pregnancy. Smoking cessation is highly encouraged by doctors during pregnancy as well as prior to pregnancy. For both doctors and pregnant women, it is important to know whether exposure to tobacco smoking before and/or during pregnancy could differentially impact the offspring, and how. The objective of this work was to identify and characterize alterations in placental DNA methylation associated with cigarette consumption in women who actively smoked during their pregnancy as well as in former smokers who quit at least 3 months prior to their pregnancy. Placenta samples from 568 women were analyzed using the Illumina HM450k BeadChip. A multidisciplinary approach combining statistical analyses with biological knowledge of the epigenome landscape enabled the identification and characterization of specific genomic regions differentially methylated in the placentas at birth as a result of cigarette smoking exposure during or prior to pregnancy (Fig. 1).

## Methods

### Aim

This work aimed at identifying cigarette-induced alterations in placental DNA methylation in women currently smoking during pregnancy and in women who quit in anticipation of pregnancy.

### Study population

Study participants included in this analysis are a subset of the participants enrolled in the EDEN Mother-Child Cohort between 2003 and 2006 [34]. EDEN is a two-center study that included 2002 pregnant women, mainly Caucasian, before 24 weeks of gestation in the university hospitals of Nancy and Poitiers, France. The exclusion criteria were multiple pregnancies, pre-pregnancy diabetes, French illiteracy, and plans to move outside the region within the following 3 years. Lifestyle, demographic, and medical data were collected by questionnaires and interviews during pregnancy and after delivery. Paternal smoking status at conception and during pregnancy was collected by questionnaire (most fathers answered at the beginning of trimester 2). Maternal passive smoking exposure (at home, at work, or anywhere else) at each trimester of pregnancy was collected by questionnaire after birth and considered as not exposed, exposed through the whole pregnancy, intermittently exposed, or missing information. DNA methylation (DNAm) was measured in placental samples from 668 women [35]. One biopsy was collected at the fetal side of each placenta at delivery by the midwife or the technician of the study using a standardized procedure [35]. Samples of around 5 mm^3^ were excised a few centimeters from the insertion of the umbilical cord under the chorio-amniotic membrane, washed in a saline solution, and were immediately frozen at − 80 °C. The protocol was similar for both centers. The EDEN cohort received approval from the ethics committee (CCPPRB) of Kremlin Bicêtre and from the French data privacy institution *Commission Nationale de l’Informatique et des Libertés* (CNIL). Written consent was obtained from the mother for herself and for the offspring.

### Maternal active smoking exposure variables

Prenatal maternal cigarette smoking was collected by questionnaires administered by the midwives during prenatal and postpartum clinical examinations. *Non-smokers* were defined as women who did not smoke during the 3 months before and during pregnancy. *Current smokers* were defined as mothers smoking ≥ 1 cigarette per day throughout the duration of the pregnancy. All current smokers during pregnancy also smoked during the 3 months before pregnancy. *Former smokers* were defined as women who reported smoking during the 3 months preceding the pregnancy and declared not smoking throughout the duration of the pregnancy. Women who quit smoking during pregnancy or cases with missing information regarding their smoking status at some point during the 3 months preceding the pregnancy or during the pregnancy were excluded, leaving 568 participants for this analysis. The choice of a 3-month exposure time period prior to pregnancy was based on a compromise between a realistic and accurate assessment of the smoking status of women around conception time (minimizing measurement error due to recall bias) and a duration that would allow enough time for a large contingent of lung epithelial cells to renew [36]. This information was collected by a questionnaire administered in mid-pregnancy using the following question “Did you smoke during the 3 months preceding your pregnancy?”

### Placental DNA methylation measurements and quality control

DNA from placental samples was extracted using the QIAsymphony instrument (Qiagen, Germany). DNA concentration was determined by Nanodrop measurement and fluorescent quantification using Picogreen. No sample was discarded due to low DNA concentration. The DNA methylation analysis was performed by the Centre National de Recherche en Génomique Humaine (CNRGH, Evry, France). The DNA samples were plated onto 96-well or 48-well plates. In total, nine plates including 64 chips were used. These plates were analyzed in 4 batches. The ratios for sex (boy/girl) and recruitment center (Poitiers/Nancy) were balanced for each chip. Fifteen samples were measured in quadruplicates and one sample in duplicate across batches, sample plates, and chips to detect technical issues such as batch effects. The Illumina’s Infinium HumanMethylation450 BeadChip, representing over 485,000 individual CpG sites, was used to assess the levels of methylation in placenta samples following the manufacturer’s instructions (Illumina, San Diego, CA, USA). Raw signals of 450K BeadChips were extracted using the GenomeStudio® software (v2011.1. Illumina). The DNA methylation level of each CpG was calculated as the ratio of the intensity of fluorescent signals of the methylated alleles over the sum of methylated and unmethylated alleles (*β* value). All samples passed initial quality control and had on average more than 98% of valid data points (detection *p* value < 0.01). A refined version of the subset quantile normalization (SQN) pipeline [37] including a revised annotation file [38] was used for data processing, correction, and normalization. Data processing and normalization did not change the density distribution of the DNA methylation levels (results not shown). Intensity values were corrected for potential biases in fluorescent dye intensity and background corrected using the *lumi* R package [39] as implemented in the SQN pipeline. Probes potentially influenced by SNPs underlying the entire sequence of the probe (+ 1 or + 2 bases depending on the Infinium probe type) that are present in the EUR population of the 1000 Genome Project (http://www.1000genomes.org) at a frequency of more than 5% were removed from the analysis. Probes previously reported to map to several genomic regions were removed [40]. The SQN pipeline uses the intensity signals of high-quality (i.e., low detection *p* value) Infinium I probes as “anchors” to estimate a reference distribution of quantiles for probes in a biologically similar context based on the annotation file [37]. This reference was then used to estimate a target distribution of quantiles for InfII probes as a means to provide an accurate normalization of InfI/InfII probes and correct for the shift. SQN is performed for each individual separately. A principal component analysis and a hierarchical clustering were applied and showed no overall difference in the methylation patterns across participant samples and replicates, so that a quantile normalization was performed for between-sample normalization. After quality control and normalization steps, 426,049 CpG sites remained for analysis. Methylation beta values ranged from 0 to 1. Data points with a detection *p* value > 0.01 were excluded from subsequent analyses. To reduce the influence of potential outliers, we excluded data points below the 25th percentile minus 3 interquartile ranges or above the 75th percentile plus 3 interquartile ranges for each probe, which removed 0.4% of all methylation beta values across participants. CpGs with more than 25% of missing data were removed, leaving 425,878 CpG sites for statistical analyses.

In addition, global methylation was evaluated by measuring methylation in four CpG sites of repetitive *Alu* elements (*Alu*) and long interspersed nucleotide elements 1 (*LINE-1*) using a previously published pyrosequencing methylation assay [41]. We then used the median methylation level of the four CpG sites. Methylation values ranged from 0 to 1.

### Cellular heterogeneity of placenta samples

The cellular composition of biological samples is a potential confounder in epigenetic epidemiological studies. In the absence of reference methylomes for placental tissue, we used a reference-free method, the RefFreeEWAS package available in R [42], to estimate the cellular heterogeneity from DNA methylation array data. The method relies on the identification of latent variables as surrogates for the cell-type mixture. From the 10,000 most variable CpGs, we identified the optimal number of latent variables to be 6 and estimated the respective proportions of these cell-type surrogates in each sample. The contribution of each of the 6 latent variables and the Pearson correlation among these variables is described in Additional file 2: Fig. S1.

### Analytical approach

We hypothesized that maternal cigarette smoking during or before pregnancy could alter the placental function through modification of DNA methylation. Therefore, we investigated the relationship of maternal cigarette smoking with global DNA methylation and gene-specific methylation (Fig. 1). In order to identify potentially relevant changes in genomic methylation sites, we performed an epigenome-wide association study (EWAS) and identified differentially methylated regions (DMR). We then focused our subsequent analyses on these smoking-associated DMRs and corresponding CpGs. We first characterized the epigenetic context of the regions whose DNA methylation patterns were significantly affected by cigarette smoking. Second, we classified the DMRs and CpGs into two categories labeled as “reversible” and “memory.” Finally, we looked for proximity and/or overlaps between the DMRs and the known imprinting control regions and promoter regions in order to assess the potential impact of tobacco exposure-related methylation alterations of our DMRs on the regulated expression of these genes (Fig. 1).

### Statistical analysis for the EWAS and identification of DMRs

We studied the association between maternal smoking status (non-smoker, current, former) and the repetitive elements *Alu* and *LINE-1*, the mean and median DNA methylation level derived from all individual CpGs, and the CpG-specific methylation level using the following robust linear regression model in order to account for potential outliers and heteroscedasticity:

1 $$ {Y}_{ij}={\beta}_{0j}+{\beta}_{1j}^T\ {\mathrm{smoking}}_i+{\beta}_{2j}^T{Z}_i $$

where *Y*_*ij*_ is the methylation measurement for CpG or repetitive element *j* in subject *i*, *β*_0_ is the intercept, *β*_1_ is the vector of the unknown parameters of the smoking status of subject *i* (smoking_*i*_, coded as a categorical variable), and *β*_2_ is the vector of the unknown parameters of the set of a priori selected adjustment factors (*Z*_*i*_) including child sex, parity (0, 1, ≥ 2 children; categorical covariate), maternal age at end of education (≤ 18, 19–20, 21–22, 23–24, ≥ 25 years; categorical covariate), season of conception (categorical covariate), study center (Poitiers and Nancy), maternal body mass index (BMI) before pregnancy (≤ 18.5, 18.5–25, 25–30, ≥ 30; categorical covariate), maternal age at delivery (linear and quadratic terms), gestational duration (linear and quadratic terms), paternal smoking status at conception (non-smoker, smoker, missing information; categorical covariate), technical factors related to the methylation measurements (batch, plate, and chip coded as categorical covariates), and estimated cellular heterogeneity (continuous covariates, excluding the 6th latent variable). Adjustment factors were identified a priori as possibly associated with placental DNA methylation and/or maternal tobacco smoking during pregnancy. Missing values for paternal smoking status were modeled as a specific category so that we did not lose these observations. We applied the Benjamini and Hochberg false discovery rate (FDR) correction to the *p* values to account for multiple testing [43]. The FDR-corrected *p* values were calculated for the 425,878 CpGs for the agnostic EWAS. An FDR-corrected *p* value < 0.05 was considered statistically significant. The inflation of *p* values for the EWAS was estimated using both the genomic inflation factor (lambda), which has been widely used in GWAS [44], and the Bayesian inflation factor (BIF), which has been shown to be a more relevant approach for EWAS [45].

To identify DMRs from the EWAS results (425,878 CpGs), we used comb-p, a method relying on the Stouffer-Liptak-Kechris correction that combines adjacent CpG *p* values in sliding windows while accounting for spatial auto-correlation across the genome [46]. The significance of regional enrichment is adjusted for multiple testing. First, the *p* value of each probe is adjusted according to the correlation of this probe with its neighbors and is corrected for false discovery rate. Differentially methylated regions are then identified, and the corresponding *p* values are calculated based on correlation (Stouffer-Liptak-Kechris (SLK) *p* value). DMR *p* values were adjusted for multiple testing using the Šidák correction [47]. A DMR was considered significant when it included at least 2 probes within a window of 2000 bp and the corrected *p* value was < 0.05.

Sensitivity analyses adjusting for paternal smoking status during pregnancy or maternal passive smoking exposure rather than paternal smoking at conception were also conducted.

We compared our results from the EWAS and regional analysis with previous findings from studies investigating the relationship between maternal smoking in pregnancy and placenta and cord blood DNA methylation changes with the Illumina HM450k BeadChip.

All analyses were performed using the statistical software R (version 3.0.1) (R Core Team, 2013) and Python (version 2.7.14).

### Characterization of epigenetic context of smoking-associated DMRs

We investigated whether the smoking-associated DMRs could be associated with specific features in their global epigenomic landscape. By characterizing the epigenome environment associated with the regions most sensitive to tobacco exposure, our aims were first to generate hypotheses explaining the high sensitivity of these regions, and second to explore the biological consequences of methylation alterations on the epigenomic control of placenta development. To address these questions, we first had to identify a set of randomly selected genomic regions. The first step consisted of randomly distributing the DNA methylation levels between the participants of our study. The second step consisted of performing EWAS and DMRs analyses using the same pipeline as with the original data, leading to the selection of the first set of randomly selected regions. In order to obtain a sufficient number of random regions, steps 1 and 2 were re-iterated 50 times. This process identified 420 regions, which actually were selected by chance. Although this was not its objective, this procedure could be considered as a permutation test under the null hypothesis that “the number of identified regions is not associated with smoking status.” The empirical *p* value of this permutation test was calculated on the basis of the number of regions selected by each iteration. This empirical *p* value is less than 1 plus the number of iterations selecting a number of regions larger than the number of smoking-associated DMRs divided by the total number of iterations.

We then looked for the epigenetic marks normally associated with these regions in the placenta by using the corresponding ENCODE ChIPseq data (https://www.encodeproject.org [48]). We compared the enrichment of our smoking-associated DMRs to that of the 420 randomly selected regions for the following epigenetic marks: the post-translational modifications (PTM) of histone H3, mono- and trimethyl lysine 4 (H3K4me1 and me3), and the acetylation of lysine 27 (H3K27ac). The files described in Additional file 1: Table S1 corresponding to the read counts for the presented histone H3 PTMs were retrieved from ENCODE (www.encodeproject.org) and used to produce heatmaps and profiles to map these modifications on our smoking-associated DMRs and random regions. The deepTools software (deeptools.readthedocs.io) were used with options *computeMatrix reference-point --referencePoint TSS --binSize 50 --beforeRegionStartLength 2000 --afterRegionStartLength 2000 --sortRegions keep* to extract PTM profiles.

### Identification of reversible and epigenetic memory methylation profiles among CpGs and DMRs

**Fig. 2:**
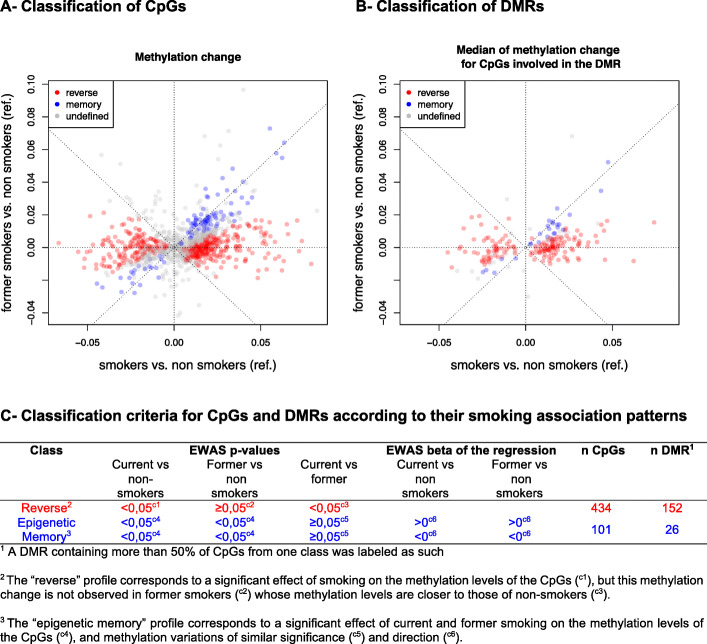
Classification of “epigenetic memory” and “reversible” CpGs and DMRs according to their smoking association methylation patterns. Plots of the regression coefficients for CpGs (**a**) and of the median of CpGs regression coefficients within each DMR (**b**) representing methylation changes in smokers versus non smokers (x-axis) and former smokers versus non smokers (y-axis). Criteria for classifying CpGs and DMRs as "epigenetic memory" or "reversible" (**c**). Results were adjusted for child sex, parity, education level, season of conception, study center, maternal body mass index before pregnancy, maternal age at delivery, gestational duration, paternal smoking status at conception, batch, plate and chip, and estimated cellular heterogeneity

Smoking-associated DMRs and corresponding CpGs were classified into categories, defined by the variations of the methylation patterns between the three groups of women, current smokers, non-smokers, and former smokers. First, by considering the regression coefficients and *p* values of the three smoking groups for each differentially methylated CpG, we labeled each CpG according to its pattern of association with smoking status as “reversible” (alteration present in current smokers but absent in the other two groups) or “memory” (alteration present in current smokers as well as in former smokers, compared to non-smokers) (Fig. 2a, c). Second, each of the 203 DMRs was assigned to the category most represented among its CpG labels and corresponding to at least 50% of its CpGs (Fig. 2b, c). The other DMRs, including those containing the same proportions of CpGs with different labels, were labeled “undefined.”

### Identification of imprinting control regions potentially affected by exposure to tobacco

We then addressed the question of whether the alterations of the DNA methylation patterns induced by cigarette smoking could have consequences on the regulation of the expression of imprinted genes. As opposed to most genes, which show bi-allelic expression (from both paternal and maternal alleles), imprinted genes are expressed only on one of the two parental alleles. This monoallelic expression is known to be controlled by the allelic differential epigenetic marks, often DNA methylation, on regions nearby or more distant, known as imprinting control regions (ICR). In humans, approximately 300 imprinted genes have been identified so far, which tend to group in clusters with shared ICR. Presently, 246 and 166 imprinted genes are respectively recorded in www.geneimprint.com and igc.otago.ac.nz databases, with 102 genes common to the two databases. Placenta-imprinted genes have also been identified and recorded by Yuen et al. [49] and Hamada et al. [50]. In order to assess the potential impact of methylation alterations in our smoking-associated DMRs on the regulated expression of these genes, we looked for proximity and/or overlaps between our smoking-associated DMRs and these known imprinted gene loci. The candidate imprinted gene loci were defined by systematically matching the genes of the Illumina HM450k BeadChip annotations corresponding to smoking-associated DMRs and the gene annotations present on databases *MetaImprint* (*http://202.97.205.76:8080/MetaImprint/* [51]), *geneimprint* (*http://www.geneimprint.com* [52]), and *igc.otago* (*http://igc.otago.ac.nz* [53]) as well as the imprinted gene loci defined by Yuen et al. [49] and Hamada et al. [50]*.*

## Results

### Population characteristics

The participating mothers had a median age of 29 (interquartile range (IQR) = [26, 32]) years old, with a median pre-pregnancy body mass index of 22 (IQR [20, 25]) kg/m^2^ (Table 1). The median gestational duration was 40 (IQR [39, 41]) weeks, and 30 babies (5.3%) were born preterm (< 37 gestational weeks). Among the 568 women participating in this analysis, 381 (67.1%) were non-smokers (i.e., did not smoke in the 3 months before pregnancy nor during the pregnancy), 117 (20.6%) were current smokers (i.e., did smoke ≥ 1 cigarette per day throughout the duration of pregnancy), and 70 (12.3%) were former smokers (i.e., did smoke in the 3 months preceding the pregnancy and stopped before the pregnancy). The prevalence of paternal smoking at conception was 40%. The estimated cellular heterogeneity differed according to the smoking status (Table 1).

**Table 1:** Characteristics of the EDEN study population (*n* = 568)

**Characteristics**	**All (*n*** **= 568),** ***n*** **(%)**	**Non-smoker (*n*** **= 381 (67%)),** ***n*** **(%)**	**Former smoker (*n*** **= 70 (12%)),** ***n*** **(%)**	**Current smoker (*n*** **= 117 (21%)),** ***n*** **(%)**	***p*** value*
**Center**					< 0.01
Poitiers	231 (41)	140 (37)	25 (36)	66 (56)	
Nancy	337 (59)	241 (63)	45 (64)	51 (44)	
**Sex of offspring**					0.24
Male	293 (52)	206 (54)	33 (47)	54 (46)	
Female	275 (48)	175 (46)	37 (53)	63 (54)	
**Parity**					0.71
0	239 (42)	155 (41)	35 (50)	49 (42)	
1	226 (40)	156 (41)	24 (34)	46 (39)	
≥ 2	102 (18)	69 (18)	11 (16)	22 (19)	
Missing	1 (0)	1 (0)			
**Maternal age at the end of education (years)**					< 0.01
≤ 18	105 (18)	51 (13)	12 (17)	42 (36)	
19–20	89 (16)	58 (15)	11 (16)	20 (17)	
21–22	127 (22)	91 (24)	12 (17)	24 (21)	
23–24	134 (24)	105 (28)	14 (20)	15 (13)	
≥ 25	113 (20)	76 (20)	21 (30)	16 (14)	
**Season of conception**					0.50
January to March	120 (21)	80 (21)	15 (21)	25 (21)	
April to June	129 (23)	89 (23)	19 (27)	21 (18)	
July to September	157 (28)	110 (29)	18 (26)	29 (25)	
October to December	162 (29)	102 (27)	18 (26)	42 (36)	
**Pre-pregnancy body mass index (kg/m**^**2**^**)**					0.08
< 18.5	47 (8)	26 (7)	5 (7)	16 (14)	
18.5–25	385 (68)	258 (68)	52 (74)	75 (64)	
25–30	94 (17)	65 (17)	12 (17)	17 (15)	
≥ 30	34 (6)	25 (7)		9 (8)	
Missing	8 (1)	7 (2)	1 (1)		
**Preterm births**	30 (5)	20 (5)	4 (6)	6 (5)	0.98
**Paternal smoking at conception**					< 0.01
No	265 (46.7)	233 (61.2)	17 (24.6)	15 (12.8)	
Yes	220 (38.8)	100 (26.2)	41 (59.4)	79 (67.5)	
Missing	82 (14.5)	48 (12.6)	11 (15.9)	23 (19.7)	
**Paternal smoking during pregnancy**					< 0.01
No	285 (50)	247 (65)	24 (34)	14 (12)	
Yes	206 (36)	91 (24)	36 (51)	79 (68)	
Missing	77 (14)	43 (11)	10 (14)	24 (21)	
**Maternal passive smoking during pregnancy**					< 0.01
No	365 (64)	300 (78.7)	45 (64.3)	20 (17.1)	
Yes	158 (28)	51 (13.4)	18 (25.7)	89 (76.1)	
Missing/intermittently exposed	45 (8)	30 (7.9)	7 (10.0)	8 (6.8)	
	***Continuous covariates***				
	**Median [IQR]**	**Median [IQR]**	**Median [IQR]**	**Median [IQR]**	
**Maternal age (years)**	29 [26, 32]	30 [27, 33]	28 [26, 32]	27 [24, 31]	< 0.01
**Gestational duration (weeks)**	40 [39, 41]	40 [39, 41]	40 [39, 41]	40 [39, 41]	0.23
**Birthweight (g)**	3330 [3030, 3630]	3360 [3070, 3670]	3330 [3070, 3657]	3230 [2920, 3420]	< 0.01
**Estimated cellular heterogeneity**					
1st covariate	0.14 [0.05, 0.22]	0.14 [0.04, 0.22]	0.12 [0.03, 0.21]	0.15 [0.09, 0.22]	0.10
2nd covariate	0.15 [0.06, 0.24]	0.15 [0.06, 0.24]	0.17 [0.09, 0.25]	0.15 [0.05, 0.25]	0.57
3rd covariate	0.34 [0.17, 0.46]	0.35 [0.17, 0.47]	0.33 [0.18, 0.42]	0.31 [0.17, 0.46]	0.54
4th covariate	0.11 [0.06, 0.18]	0.10 [0.05, 0.16]	0.13 [0.08, 0.19]	0.13 [0.07, 0.19]	0.01
5th covariate	0.06 [0.01, 0.12]	0.06 [0.01, 0.12]	0.06 [0.00, 0.10]	0.09 [0.03, 0.13]	0.05
6th covariate	0.11 [0.04, 0.21]	0.11 [0.05, 0.24]	0.11 [0.05, 0.22]	0.09 [0.04, 0.16]	0.11

*Kruskal-Wallis test for continuous covariates and chi-square test for categorical covariates

### Maternal cigarette smoking is associated with lower *LINE-1* methylation levels

**Fig. 3:**
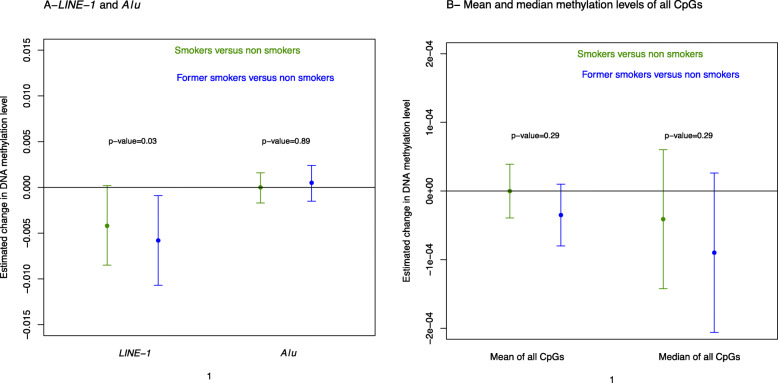
Adjusted association between smoking status and repetitive DNA methylation elements (**a**) and mean and median methylation levels of all CpGs present on the 450K beadchip (**b**). Dots represent the regression coefficients, and bars represent the 95% confidence intervals of the association between smoking status during pregnancy and methylation levels measured in 568 placental biopsies of the EDEN cohort. Results were adjusted for child sex, parity, education level, season of conception, study center, maternal body mass index before pregnancy, maternal age at delivery, gestational duration, paternal smoking status at conception, batch, plate and chip, and estimated cellular heterogeneity

The average methylation level was 0.26 (± 0.02) for *LINE-1* and 0.16 (± 0.01) for *Alu*. Women who smoked had significantly lower methylation levels for *LINE-1* compared to non-smoking women, with a slightly larger association for former smokers (− 0.006 (− 0.011; − 0.001)) than for current smokers (− 0.004 (− 0.008; 0.001)) (Fig. 3a). No significant effect was observed for *Alu* elements nor for the mean or median methylation levels derived from all individual CpGs present on the 450K beadchip (Fig. 3b).

### Genomic regions are differentially methylated according to maternal smoking

**Fig. 4:**
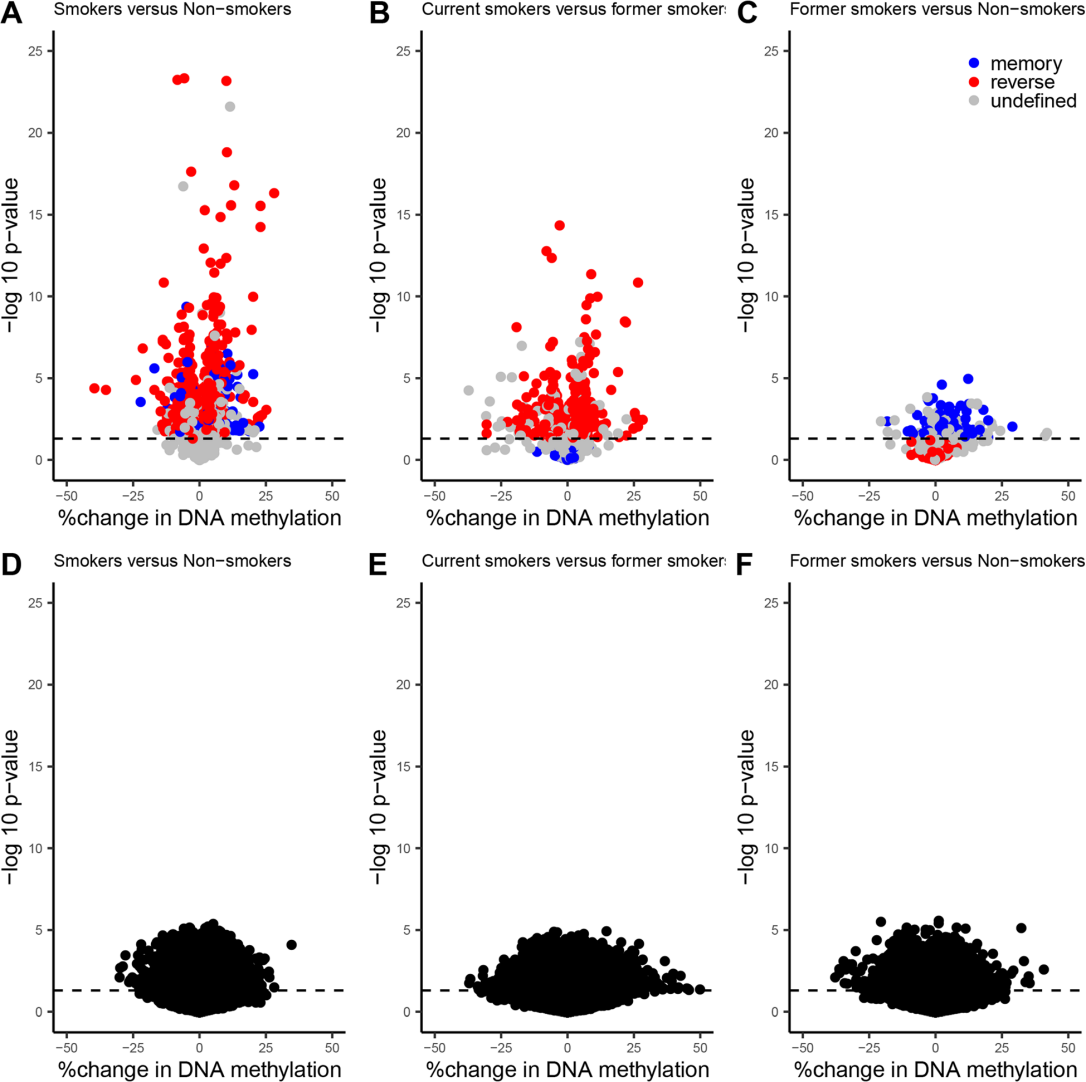
Volcano plots showing the percent change in DNA methylation of each of the 1023 CpGs included in the 203 DMRs (**a**, **b**, **c**) and of each CpG not significantly associated with smoking status (**d**, **e**, **f**) as a function of the nominal *p* value (uncorrected *p* value of the EWAS) of each CpG. Three differential analyses are shown : smokers versus non-smokers (**a** and **d**), smokers versus former smokers (**b** and **e**) and former smokers versus non-smokers (**c** and **f**). Results were adjusted for child sex, parity, education level, season of conception, study center, maternal body mass index before pregnancy, maternal age at delivery, gestational duration, paternal smoking status at conception, batch, plate and chip, and estimated cellular heterogeneity

Among the 425,878 CpGs explored in the adjusted EWAS (Additional file 1: Table S2), 203 DMRs were identified (Additional file 1: Table S3). When we independently tested the adjusted effect of tobacco smoking on each of the 425,878 CpGs, the *p* value distribution was close to the theoretical distribution as indicated by the BIF value of 1.06 which was substantially smaller compared to the lambda (i.e., genomic inflation factor) value of 1.61 (Additional file 2: Fig. S2). These 203 DMRs included a total of 1023 CpGs (242 of which were individually significant (FDR *p* value < 0.05) in the EWAS, Additional file 1: Table S3). In addition, the number of regions selected by each of the fifty iterations of the permutation test (see the “Methods” section) varied from 0 to 138, with an average of 8.7, and none of the fifty iterations selected more than 203 regions. Based on this observation, the number of smoking-associated DMRs, 203, was significantly different from that of random regions (empirical *p* value < 0.02). We focused our subsequent analyses on these 203 DMRs and their associated 1023 CpGs. There were 54% (former smokers versus non-smokers), 84% (current smokers versus former smokers), and 87% (current smokers versus non-smokers) of CpGs whose change of DNA methylation (relative to the mean of each CpG) was higher than 1% in absolute value (Additional file 1: Table S3). Volcano plots show the percent change in DNA methylation of each of the 1023 CpGs included in the 203 DMRs as a function of the nominal *p* value, in each of the 3 comparisons between the smoking groups (Fig. 4).

Sensitivity analyses adjusting for paternal smoking status during pregnancy or maternal passive smoking exposure rather than paternal smoking at conception identified 203 DMRs (including 1035 CpGs) and 68 DMRs (including 358 CpGs), respectively. Among these DMRs, 89% when adjusting for paternal smoking status during pregnancy (181 DMRs among 203) and 90% when adjusting for maternal passive smoking exposure (61 DMRs among 68) overlapped with the initial 203 DMRs (adjusting for paternal smoking at conception), showing the robustness of the results (Additional file 1: Table S3).

### Smoking-associated DMRs are depleted in gene promoters and harbor enhancer-like features

**Fig. 5:**
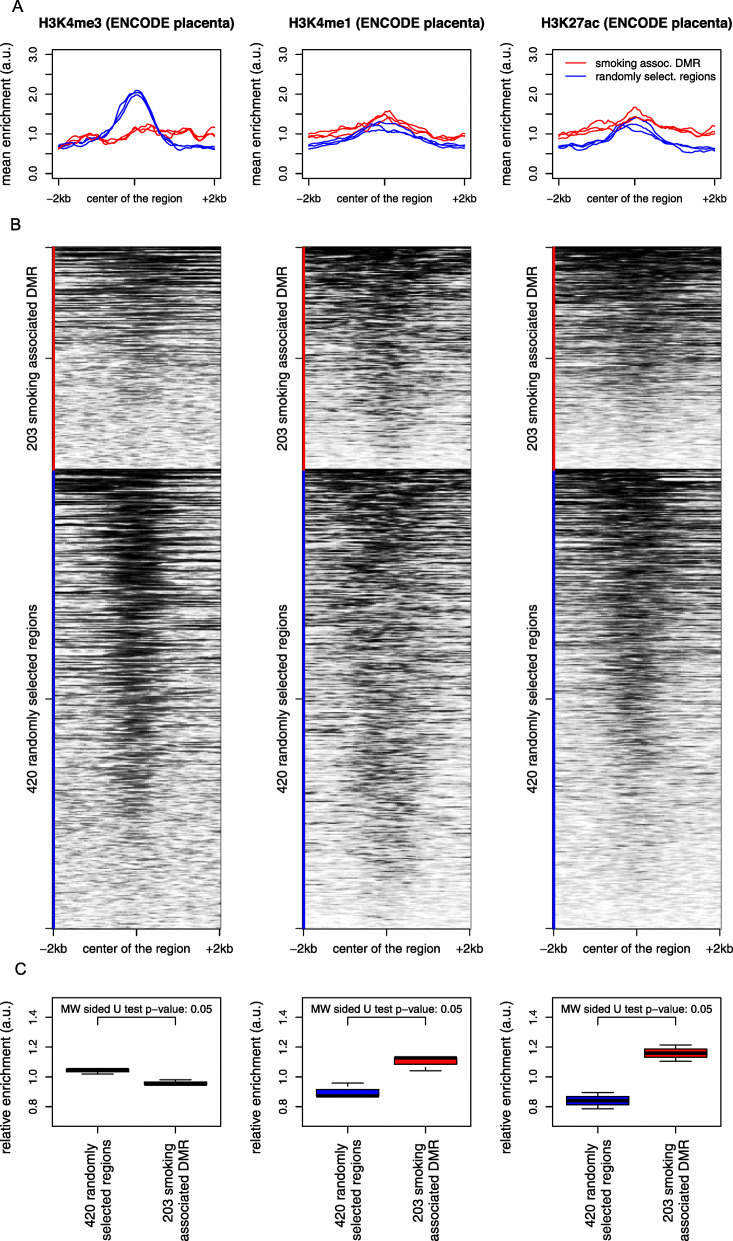
Analysis of the placenta ChIP-seq ENCODE data. The 203 DMRs associated with pregnancy smoking status are depleted in H3K4me3 and enriched in H3K4me1 and H3K27ac, compared to 420 randomly selected regions^**#**^ on the Illumina array. The curves (**a**) show the mean profiles of ChIP-seq read counts in our 203 DMRs (red) and in the 420 random regions (blue) in triplicates. The heatmaps (**b**) correspond to the ChIP-seq data of one representative placenta sample for each mark. The box plots (**c**) show the distribution of areas under the corresponding curves. The values used for measuring the relative enrichments in histone acetylation and methylation marks of our DMRs are the mean of the mean enrichment over the region centered on the DMR ± 2 kb. A non-parametric comparison was performed using a Mann and Whitney sided *U* test. Heatmaps corresponding to all three samples are shown in supplemental files (Additional file 2: Fig. S3). ^#^The “420 randomly selected regions” were obtained by running comb-p to select regions based on the null hypothesis “the number of identified regions is not associated with smoking status.” First, the DNA methylation levels were randomly redistributed, and then the analysis was performed using the same pipeline and parameters as with the real test groups, leading to the identification of CpGs and regions associated with smoking status by chance. In order to obtain a sufficient number of these random regions, the procedure was re-iterated 50 times

The analysis of ENCODE placental data comparing specific epigenetic marks between the 203 smoking-associated DMRs and 420 randomly selected regions revealed a relative depletion in H3K4me3, which is considered a hallmark of gene promoter regions, as well as a relative enrichment in the H3K4me1 and H3K27ac in our smoking-related DMRs (Fig. 5, Additional file 2: Fig. S3).

### DMRs bear “epigenetic memory” of maternal smoking before pregnancy

A total of 152 DMRs were bearing “reversible” alterations, only found in the current smokers group, whereas 26 DMRs showed alterations of placental DNA methylation not only in current smokers but also in former smokers whose placenta had not been exposed directly to cigarette smoking (Fig. 2). The latter DMRs were therefore labeled as “epigenetic memory.”

### DMRs sensitive to smoking exposure were enriched in imprinted genes

**Fig. 6:**
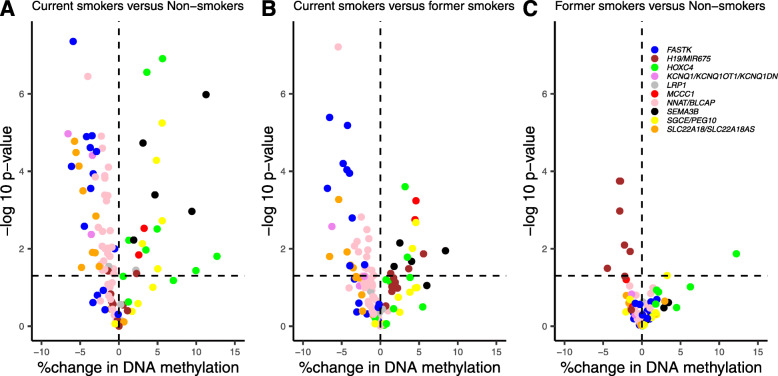
Volcano plots showing the percent change in DNA methylation of each CpG included in the DMRs found near and/or overlapping with the 10 imprinted genes clusters (16 genes) as a function of the nominal *p* value (uncorrected *p* value of the EWAS). Three differential analyses are shown: smokers versus non-smokers (**a**), smokers versus former smokers (**b**) and former smokers versus non-smokers (**c**). Results were adjusted for child sex, parity, education level, season of conception, study center, maternal body mass index before pregnancy, maternal age at delivery, gestational duration, paternal smoking status at conception, batch, plate and chip, and estimated cellular heterogeneity

The 203 smoking-associated DMRs were found near and/or overlapping with 10 imprinted genes containing loci (including 16 imprinted genes) or their imprinting control regions (ICR). As shown by the volcano plots (Fig. 6), for all CpGs within each imprinted locus, we observed a consistent variation in methylation levels, either increasing or decreasing in smokers. There are approximately 300 predicted or validated imprinted genes in the human genome (considering *geneimprint (**http://www.geneimprint.com* [52]) and *igc.otago* (*http://igc.otago.ac.nz* [53]) databases as well as by Yuen et al. [49] and Hamada et al. [50]), which represent approximately 1.2% of the ~ 25,000 genes of the human genome. By comparison, our 203 DMRs contain a total of 356 genes, including 16 imprinted genes, which therefore represent 4.5% of the genes of our DMR, demonstrating a significant enrichment in imprinted genes (*χ*^2^ *p* value of 1.0e−07) in genome regions where smoking-induced altered methylation events were observed.

**Fig. 7:**
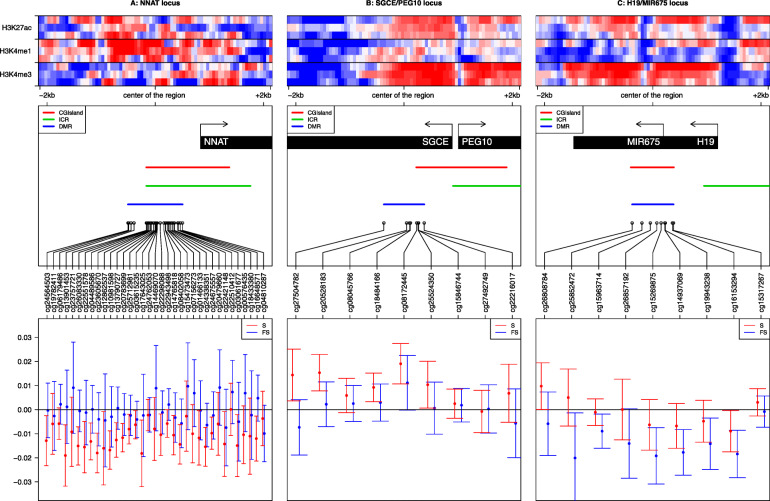
The methylation levels of the three imprinted loci are consistently modified following exposure to cigarette smoking. The left, center, and right panels are respectively centered on the DMRs associated with the *NNAT* locus ± 2 kb, the *SGCE/PEG10* locus ± 2 kb, and the *H19/MIR675* locus ± 2 kb. The top panels show the heatmaps corresponding to H3K4me3, H3K4me1, and H3k27ac enrichments in the placenta around the center of the region of interest ± 2 kb. For each mark, we downloaded triplicates from ENCODE data (see the “Methods” section). We computed the enrichment matrices from bigWig files using the deepTools software and displayed it using a custom R script. The middle panels show genes and CpG islands from the Illumina Infinium Human Methylation 450K BeadChip annotations, ICR from Pervjakova et al. [54], the DMR we identified, and the corresponding Illumina probes of the region of interest. The lower panels show the methylation changes in smokers (S) in red (resp. former smokers (FS) in blue) compared to non-smokers. The points represent the beta of the linear model, and the error bars correspond to 2 standard deviations. The *Y*-axis represents the distributions of the variations of the regression coefficient (i.e., the associated change in DNA methylation level between 0 and 1) for each CpG within the DMRs selected by our analysis

An analysis of the expression patterns of these genes in normal tissues (Additional file 3: Table S5, Additional file 2: Fig. S4) shows that most of these genes have a relatively high expression in the placenta, supporting their important role during placenta development. Three of the 203 DMRs whose methylation levels were altered by exposure to smoking were not only overlapping the imprinted gene loci but also consistently close to (< 1 kb) or overlapping the imprinted control region (known ICR) (Fig. 7). The first two loci, *NNAT/BLCAP* (20q11.23) and *SGCE/PEG10* (7q21.3), were both found associated with a reversible alteration of DNA methylation, which was respectively decreased or increased for all CpGs (but 2 for *NNAT/BLCAP*) in women currently smoking during pregnancy, compared to both former and non-smokers. *NNAT/BLCAP* was the longest DMR identified with 35 CpGs. Interestingly, the third locus, *H19/MIR675* (11p15.5), was affected by a decreased methylation not only in the placenta of currently smoking pregnant women, but methylation alterations were also detectable in the placenta from former smokers that had never been directly exposed to cigarette smoking. The 7 other DMRs were also overlapping imprinted gene loci (Additional file 2: Fig. S5).

Our results regarding the reversible/epigenetic memory status of smoking-associated DMRs and the enrichment of these DMRs in enhancer-like features and imprinted genes were robust to adjustment for paternal smoking during pregnancy or maternal passive smoking (rather than paternal smoking at conception) (not shown). Of note, the *SGCE/PEG10* (7q21.3) locus was not present in the 68 DMRs identified when adjusting for maternal passive smoking (Additional file 1: Table S3).

## Discussion

The present study identified 203 genomic regions that were significantly differentially methylated in the placenta according to cigarette smoking during pregnancy. Interestingly, 26 of these DMRs were characterized by persistent methylation changes in the placentas of former smokers, despite an absence of direct exposure of these placentas to tobacco, suggesting the possibility of an “epigenetic memory” of exposure to cigarette smoking before pregnancy. This result is also supported by the observation of a significant demethylation of *LINE-1* sequences in the placentas of former smokers. Exploration of the epigenetic status of all DMRs revealed that genomic regions bearing “enhancer-like” epigenetic marks, namely histone post-translational modifications H3K4me1 and H3K27ac, are enriched among our 203 DMRs, suggesting that placenta enhancer genomic regions could be particularly sensitive to tobacco exposure. Furthermore, tobacco-associated DMRs also overlap with genomic regions controlling imprinted genes, known to have an important role in fetal and placental development.

Potential limitations of our study relate to the characterization of the smoking phenotype. Indeed, maternal smoking was evaluated by questionnaires administered by midwives involved in the study, which might lead to an underestimation of the number of smoking women and the effect of smoking, due to under-reporting of tobacco consumption. However, on a subsample of 100 women whose cotinine levels were measured in the urine between 24 and 28 gestational weeks [55], we found only one former smoker with cotinine levels potentially compatible with current smoking (> 50 ng/mL) suggesting that self-reporting was a reasonably accurate indicator of the maternal smoking status in our cohort. Another limitation inherent to this study concerns the relatively small size of the former smokers group, which could result in a lack of statistical power and affect the significance of CpG methylation differences between current and former smokers. Such a situation could lead to a misclassification of some genomic regions altered by cigarette exposure that could be categorized in the “epigenetic memory” group while rather belonging to the “reversible” group. Finally, the group of former smokers likely includes women who stopped smoking in anticipation of pregnancy (i.e., before the conception) and women who stopped smoking in early pregnancy once they knew they were pregnant (i.e., a few days/weeks after conception). The time since quitting smoking was not available in our data. However, this might influence the methylation levels and would be an interesting piece of information to add to future research to provide insights into the existing risk faced by former smokers even months or years after cessation.

Our results showed a high lambda value of 1.61. Such inflation of low *p* values in an EWAS is not unexpected and cannot be compared with inflation commonly seen in GWAS studies. One likely explanation for the inflation is the somewhat small but multiple effects of tobacco smoking on DNA methylation throughout the epigenome giving rise to a large number of sites impacted by smoking. Using the recently developed Bayesian method, the inflation value was substantially smaller (1.06) and consistent with little evidence of inflation.

The placenta is a complex transient organ composed of different layers containing different cell types. In the absence of cytological data on our placenta samples, we used a reference-free method, based on latent factor models, to estimate cellular heterogeneity from DNA methylation array data. To account for the potential effect of cell types on DNA methylation levels, we adjusted for cellular heterogeneity using a latent factor method. Although this approach enabled us to account for the impact of cellular heterogeneity on DNA methylation, it is likely not sufficient to fully account for cell-type composition differences. Indeed, since this approach is data-driven, it is possible that residual confounding by cellular heterogeneity may have influenced some of our results. In particular, the real number of cell types in the placenta is expected to be higher than the 6 latent variables captured by the RefFreeEWAS algorithm, and therefore, the residual effect of cell types on placental methylation levels cannot be ruled out.

Data from the literature show that for some loci, DNA methylation variations could be related to the nearby genetic variations [56–59], which may also explain to some extent the interindividual DNA methylation variations observed in our study. However, a recent study [60] identified environmentally responsive variably methylated regions (VMRs) strongly enriched in imprinted loci, which is fully consistent with our finding that exposure to cigarette smoking, also an environmental assault, could affect methylation in imprinted gene regions. In view of these data, a possible influence of inter-individual genetic variations in this phenomenon would be minimal in comparison with the effect due to exposure to cigarette smoking.

Assumptions of linear regression are often violated in EWAS, especially the normality of the distribution of the residuals. Nonetheless, a study [61] conducted on blood samples analyzed using the EPIC BeadChip demonstrated that linear regression is a valid statistical methodology for DNA methylation studies, despite the fact that the data do not always satisfy the assumptions of the linear regression. The authors showed that CpGs with methylation levels at the extremes (i.e., approaching 0 or 1) were more likely to violate the assumptions compared to CpGs with intermediate levels of methylation. This pattern generally held for all 4 assumptions tested but was most apparent for tests of skewness and kurtosis. They further showed that the lack of normal residuals, an incorrectly specified link function, or heteroskedasticity did not lead to either false-positive or false-negative associations.

The present study includes the largest sample size of the placentas investigated to date, which were collected in the context of a longitudinal cohort with repeated data on maternal smoking before and during pregnancy. The placenta is considered as an accurate “record” of children’s in utero exposures [62] and represents only a partial barrier, since many chemicals, such as polycyclic aromatic hydrocarbons, can pass through and reach the fetus [63]. Additionally, this study is the first observation and characterization of DNA methylation alterations in the placentas of former smokers. An important specificity of the present study is the analysis of the epigenetic characteristics of the tobacco-related DMRs by exploiting external publicly available data from ENCODE. This analysis not only identified a higher sensitivity of “enhancer-like” regions to tobacco exposure but also demonstrates the biological relevance of our data. Finally, we accounted for the potential effect of paternal tobacco smoking on placental methylation and showed consistent conclusions when adjusting for paternal tobacco smoking at conception or during pregnancy and when adjusting for maternal passive smoking exposure, thus suggesting an intrauterine effect of maternal active smoking on placenta DNA methylation.

Only one study previously investigated placenta DNA methylation in relation to maternal smoking using a high-density genome-wide approach [29]. Out of the 1023 CpGs included in our 203 regions sensitive to smoking, 9 CpGs were also found associated (FDR-corrected *p* value < 0.05) with maternal smoking by Morales et al. (Additional file 1: Tables S3 S4). These 9 CpGs are located on the following genes: *TRIO*, *CMIP/PLCG2*, *TINAGL1*, *PDXK*, *ACOX3*, *TGM1*, and *PDGFB/RPL3*, and are all included in DMRs associated with a reversible methylation pattern upon smoking cessation except *PDXK* which is categorized as an epigenetic memory. *TRIO* (trio Rho guanine nucleotide exchange factor) was reported to interact with benzo(a) pyrenes, resulting in a decreased gene expression [29], which could be in accordance with the increased methylation levels observed in relation to maternal smoking exposure in both Morales et al.’s and our study. *TINAGL1* (tubulointerstitial nephritis antigen like 1) and *PDGFB* (platelet-derived growth factor subunit B) are broadly expressed in the placenta and have been identified as pro-angiogenic factors [64]. Angiogenesis is a major process in pregnancy, and *PDGFB* is likely to play an important role in the maintenance of utero-placental homeostasis [65]. Nicotine, one of the thousands of compounds of tobacco smoke, is also a pro-angiogenic factor [66]. In our results, *TINAGL1* showed lower methylation and *PDGFB* higher methylation in smokers compared to non-smokers. In a recent study, *TINAGL1* was found downregulated in the placenta of pre-eclamptic pregnant women compared to normotensive women [67]. Although further studies are required in order to determine the functional relationship between these genes and their placental methylation, our findings might be of interest in the search for explanations of the apparent protective effect of maternal smoking on the risk of pre-eclampsia, and more generally in the relationship between maternal tobacco smoking and alterations in placental angiogenesis [68].

Several studies identified CpGs altered in cord blood samples following exposure to tobacco smoking during pregnancy [23–25]. Although some of our CpGs in the placenta overlapped with the results of these studies, the direction for the associations was somewhat inconsistent (Additional file 1: Tables S2, S3). This lack of agreement between the placenta and the cord blood can be explained by the fact that placenta and cord blood cells have different epigenetic signatures reflecting their tissue differentiation [69].

An interesting observation is the demethylating effect of exposure to tobacco smoking on LINE-1 sequences. Compared to other tissues, the human placenta is known to have lower levels of *LINE-1* and *Alu* methylation [70, 71]. Interestingly, although the same approach as for *LINE-1* analysis was performed to measure methylation levels of *Alu* regions, which are also interspersed repeats, the latter did not vary significantly as a function of tobacco exposure. Furthermore, the mean/median methylation levels of the 425,878 CpGs did not show any significant association with the smoking status. Therefore, although methylation levels of *LINE-1* and *Alu* are both considered as surrogate markers for global methylation levels, the present study does not suggest that tobacco exposure induces global changes in methylation levels but rather highlights that the methylation levels of these repeats are differentially affected by tobacco exposure. Furthermore, this observation of a differential sensitivity to tobacco exposure between different genomic repeats regions is paralleled by the different sensitivities to tobacco between unique genomic regions measured by the high-resolution genome-wide methylome Illumina 450K array. Only a few studies had so far investigated global methylation in the placenta. One study of approximately 40 first trimester placentas did not show any effect of maternal smoking on neither *AluYb8* nor *LINE-1* methylation levels [72]. In another study of 379 term placentas, *AluYb8* methylation level was significantly higher in smoking women compared to non-smokers, while no evidence of significant association was found for *LINE-1* [73]. Another study conducted on 96 term placentas failed to find any association between *LINE-1* methylation and maternal smoking [74]. These apparent discrepancies with our results could be explained by the lack of statistical power to detect an effect of smoking for the first study and for the other two the fact that the samples were collected preferentially on the maternal side of the placenta, as opposed to our study conducted on samples representing the fetal side of the placenta. While the overall difference of DNA methylation observed in our study is relatively small compared to cancer-related DNA methylation variations, it is in the same range as to what has been observed in other epidemiological studies. It is also possible that there is heterogeneity between *LINE-1* elements in terms of demethylation levels as suggested by previous observations in cancer [75].

Although a few studies have investigated the effect of time since quitting smoking on blood DNA methylation [76, 77], the potential effect of past smoking on the methylation of the placenta had not been explored. We focused on women who had smoked but quit smoking before their pregnancy and therefore before the differentiation of the placenta. Our results identified 152 DMRs where the DNA methylation profiles were altered in the placenta of women currently smoking during their pregnancy and not in the placenta of non-smokers or former smokers, suggesting that the DNA methylation alterations of these regions could be associated with direct exposure of the placenta to tobacco, and “reversible” upon smoking cessation. Another 26 regions showed altered DNA methylation profiles in the placenta not only in women actively smoking during their pregnancy but also in women exposed to cigarette smoking prior to their pregnancy. This result, in line with the significant demethylation of *LINE-1* sequences observed in the placenta of former smokers, suggests that the placentas of former smokers bear epigenetic marks reflecting a past exposure to smoking, prior to the development of the placenta. It implies that somehow the memory of past smoking is transmitted to the placentas that have not been directly exposed to smoking. This observation raises new fundamental questions such as the mechanisms and molecular basis involved in such a transmission, which are presently totally unknown. Indeed, although the memory of past exposure to tobacco could involve alterations of epigenetic marks somehow transmitted to the oocyte, which would remain in the zygote and through the differentiation of placenta cells, it is possible and likely that non-epigenetic mechanisms would be involved. For instance, past smoking could affect the histological features of both the maternal and the fetal components of the placenta through the influence of maternal factors either via direct cellular contact or through endocrine signaling.

Since DNA methylation is only one of the many chemical marks involved in shaping the epigenome, we wished to explore other elements of the epigenetic landscape of the DMRs identified as associated with maternal smoking status. The question was whether some other elements of the epigenetic context of these DMRs could be associated with the particular sensitivity of these regions to smoking exposure. A meta-analysis of ENCODE data from the placenta, obtained from independent external experimental settings, enabled us to explore the epigenome landscape normally associated with the most affected genomic regions. This analysis compared our tobacco-sensitive DMRs to genome regions of similar sizes that we randomly selected by using exactly the same EWAS and DMR pipelines but starting from samples which were randomly distributed between smoking status groups. The pattern of association with ENCODE epigenetic marks of these random regions showed a predominant association with H3K4me3, a gene promoter-associated hallmark, which is expected since our analysis was performed on CpG-rich regions. In contrast, our smoking-associated DMRs were depleted in histone post-translational modification (PTM) marks normally associated with poised gene promoter regions, such as H3K4me3, but were rather enriched in PTM generally associated with “enhancer” regions, H3K4me1 and H3K27ac. This observation suggests that this epigenetic context is somehow related to a higher sensitivity of these regions to tobacco exposure. Besides its biological meaning, this evidence demonstrates the biological relevance of our data and therefore could be considered as a biological validation of the identified DMRs.

Another major observation from this work is that tobacco-induced DNA methylation alterations also affect imprinting control regions (ICR), whose allelic differential methylation normally controls the allele-specific expression of clusters of “imprinted” genes. A recent work highlighted the incomplete erasure of germline DNA methylation in the human placenta and the effect of some allele-specific DNA methylation pattern on the expression of imprinted genes in the placenta [50]. Interestingly, three of our DMR regions whose methylation profiles were altered by cigarette smoking exposure were found close to (< 1 kb) three imprinting loci, whose ICR are well defined. This finding suggests that the imprinted genes controlled by these ICR could have their expression patterns directly affected by smoking and potential mechanisms by which tobacco could impact the epigenome and fetal growth. The first two loci, *NNAT/BLCAP* (20q11.23) and *SGCE/PEG10* (7q21.3), were both associated with altered (decreased and increased, respectively) DNA methylation in women currently smoking during pregnancy and classified as “reversible” regions. Increased expression of *NNAT/BLCAP* was associated with an increased risk of large or small for gestational age infants [78]. As for *SGCE/PEG10*, its methylation in the cord blood was associated with birth weight [79, 80] and its expression in bronchial epithelium was associated with tobacco smoking in adults [81]. The demethylation of the third locus, which controls the expression of *H19/MIR675* (11p15.5), was observed not only in the placenta of currently smoking women but also detectable in the placenta of former smokers suggesting that this important imprinted locus could be part of those bearing the memory of past exposure to tobacco. Lower methylation of this gene has been found associated with increased expression in fetal growth-restricted placentas [82]. In a recent study, lower methylation of *H19* in the cord blood was found significantly associated with maternal smoking during pregnancy [83]. Furthermore, placental expression of *H19* was associated with large for gestational age infants [78], and genetic variants have been identified and related to birth weight [84, 85]. Our finding of a demethylation of the *H19/MIR675* locus in the placenta exposed to cigarette smoking, combined with previous observations, strongly supports the hypothesis that the *H19/MIR675* locus is a major determinant of fetal growth and that cord blood and placental DNA methylation are both sensitive to direct smoking exposure.

## Conclusion

This study investigating the effect of smoking on human placental DNA methylation at high resolution in the largest sample size published to date has led to the identification of DMRs in the placentas of current smokers as well as former smokers. These tobacco-induced DMRs are enriched in epigenetic marks corresponding to enhancer regions, and some of them overlap regions containing imprinted genes or controlling their monoallelic expression, suggesting mechanisms by which tobacco could impact the epigenome and affect placental development and fetal growth. Additionally, altered DNA methylation patterns were not only observed in the placenta directly exposed to cigarette smoking, but some alterations of DNA methylation patterns were also observed in the placenta of women who had smoked but quit smoking in anticipation to pregnancy, suggesting the establishment of a “memory” of exposure to tobacco and transmission of epigenetic marks to placentas that had never been directly exposed to smoking. Hence, this work brings new concepts and questions in the field of human epigenetics and suggests mechanisms by which an exposure to environmental cues could have not only direct but also long-term effects on human health. In addition to the important potential impact on our current knowledge of epigenetic transmission, our results bring essential information in terms of public health concerning potential long-term detrimental effects of smoking in young women, which could affect their offspring even after smoking cessation. This scientific report should support the primary and secondary prevention of smoking-associated health effects.

## Supplementary information

**Additional file 1: Table S1.** Metadata of analysed ENCODE files. **Table S2.** Results from the Epigenome Wide Association Study (EWAS): 1,800 CpGs differentially methylated between the three groups of women (non-smokers, current smokers or former smokers) (*p*-value corrected for False Discovery Rate (FDR) <0.05). **Table S3.** Results from the regional analysis using comb-p : 203 Differentially Methylated Regions including 1023 CpGs. **Table S4.** Comparison of the number of CpGs found significantly associated with smoking between the present EDEN study and previous studies conducted on placenta and cord blood.

**Additional file 2: Supplementary Figure S1.** cellular heterogeneity estimated by the RefFreeEWAS method on 668 placenta samples from the EDEN cohort (A) contribution (%) of each latent variable (B) Pearson correlation between the 6 latent variables. **Supplementary Figure S2.** Q-Q plot, genomic inflation factor (lambda) and Bayesian inflation factor (BIF) for the association between each methylation site of the EWAS (425,878 CpGs) and tobacco smoking during pregnancy (current, never, former smoker). Results were adjusted for child sex, parity, education level, season of conception, study center, maternal body mass index before pregnancy, maternal age at delivery, gestational duration, paternal smoking status at conception, batch, plate and chip and estimated cellular heterogeneity. **Supplementary Figure S3.** H3K4me3 (A), H3K4me1 (B) and H3K27ac (C) ChIPSeq signal of 3 placenta replicates. The top panels represent the mean signal values centered on our 203 DMRs +/-2kb (blue) and the mean signal values centered on the 420 random regions +/-2kb (green). The center panels represent heatmaps of the corresponding ChIP-Seq signals centered on our 203 DMRs +/-2kb (upper heatmaps) or on the 420 random regions+/-2kb (lower heatmaps). **Supplementary Figure S4.** Expression of genes in normal human tissues from RNA-seq data. The barplots show average expression levels of the 16 imprinted genes overlapping our DMRs in normal tissues and development stages (adult, embryonic and fetal). For the sake of clarity, the plots are separated in two panels of 8 genes. Vertical lines on the top of the barplots represent standard deviations. Barplots representing expressions in placenta are in green. RNA-seq data in normal tissues were provided by GTEx portal and NCBI Sequence Read Archive (datasets PRJNA280600, PRJEB4337, PRJEB2445, PRJNA270632, GSE70741, GSE53096). The expression levels are represented in log-transformed RPKM (Read per Kilobase Million) values after addition of a pseudo count of 1, i.e. log-transformed RPKM = log2(1+RPKM). MIR675 has no expression in any of the analyzed samples. **Figure S5.** The methylation levels of 7 imprinted loci are consistently modified following exposure to cigarette smoking. The left, center and right panels are respectively centered on the DMRs associated with the NNAT locus +/- 2kb, the SGCE/PEG10 locus +/- 2kb and the H19/MIR675 locus +/- 2kb. The top panels show heatmaps respectively corresponding to H3K4me3, H3K4me1 and H3k27ac enrichments in placenta around the center of the region of interest +/- 2 kilobases. For each mark we downloaded triplicates from ENCODE data (see Methods). We computed the enrichment matrices from bigWig files using the deepTools software and displayed it using a custom R script. The middle panels show genes and CpG islands from the Illumina Infinium Human Methylation 450K BeadChip annotations, ICR from Pervjakova et al. [72], the DMR we identified and the corresponding Illumina probes of the region of interest. The lower panels show the methylation changes in smokers (S) (resp. Former Smokers (FS)) in red (resp. blue) compared to nonsmokers. The points represent the beta of the linear model and the error bars correspond to 2 standard deviations. The Y-axis represents the distributions of the variations of the regression coefficient (i.e. the associated change in DNA methylation level between 0 and 1) for each CpG within the DMRs selected by our analysis.

**Additional file 3: Table S5.** Imprinted genes whose ICR [50, 51, 86] and/or promoters are affected by tobacco-induced altered DNA methylation profiles.

## Data Availability

The EDEN datasets analyzed in the presented study are not publicly available as they are containing information that could compromise the research participant’s privacy/consent. However, they are available from the corresponding author on reasonable request and with permission from the EDEN Steering Committee. The code used to generate the results is available upon request to the corresponding author. NCBI Dataset: We used RNA-seq data provided by the NCBI Sequence Read Archive (SRA) datasets PRJNA280600, PRJEB4337, PRJEB2445, and PRJNA270632 and NCBI GEO datasets GSE70741 and GSE53096. Genotype-Tissue Expression Project https://gtexportal.org/ (corresponding publication: https://www.ncbi.nlm.nih.gov/pmc/articles/PMC4010069/) We used RNA-seq data available on https://gtexportal.org/home/datasets. ENCODE Dataset: We used Chip-seq data provided by the ENCODE datasets https://www.encodeproject.org (corresponding publication: https://www.ncbi.nlm.nih.gov/pmc/articles/PMC3439153/).
