# Supplementary material for: Immediate and durable effects of maternal tobacco consumption alter placental DNA methylation in enhancer and imprinted gene-containing regions
Source: BMC Med. 2020 Oct 7;18:306. doi: 10.1186/s12916-020-01736-1 (PMC7542140; doi:10.1186/s12916-020-01736-1)
Supplement: Supplementary file 2 — Additional file 2: Supplementary Figure S1. cellular heterogeneity estimated by the RefFreeEWAS method on 668 placenta samples from the EDEN cohort (A) contribution (%) of each latent variable (B) Pearson correlation between the 6 latent variables. Supplementary Figure S2. Q-Q plot, genomic inflation factor (lambda) and Bayesian inflation factor (BIF) for the association between each methylation site of the EWAS (425,878 CpGs) and tobacco smoking during pregnancy (current, never, former smoker). Results were adjusted for child sex, parity, education level, season of conception, study center, maternal body mass index before pregnancy, maternal age at delivery, gestational duration, paternal smoking status at conception, batch, plate and chip and estimated cellular heterogeneity. Supplementary Figure S3. H3K4me3 (A), H3K4me1 (B) and H3K27ac (C) ChIPSeq signal of 3 placenta replicates. The top panels represent the mean signal values centered on our 203 DMRs +/-2kb (blue) and the mean signal values centered on the 420 random regions +/-2kb (green). The center panels represent heatmaps of the corresponding ChIP-Seq signals centered on our 203 DMRs +/-2kb (upper heatmaps) or on the 420 random regions+/-2kb (lower heatmaps). Supplementary Figure S4. Expression of genes in normal human tissues from RNA-seq data. The barplots show average expression levels of the 16 imprinted genes overlapping our DMRs in normal tissues and development stages (adult, embryonic and fetal). For the sake of clarity, the plots are separated in two panels of 8 genes. Vertical lines on the top of the barplots represent standard deviations. Barplots representing expressions in placenta are in green. RNA-seq data in normal tissues were provided by GTEx portal and NCBI Sequence Read Archive (datasets PRJNA280600, PRJEB4337, PRJEB2445, PRJNA270632, GSE70741, GSE53096). The expression levels are represented in log-transformed RPKM (Read per Kilobase Million) values after addition of a pseudo count of 1 [file 12916_2020_1736_MOESM2_ESM.docx]

**Title: Immediate and durable effects of maternal tobacco consumption alter placental DNA methylation in enhancer and imprinted gene-containing regions**

**Supplementary Materials**

**Authors:**

Sophie Rousseaux ([sophie.rousseaux@univ-grenoble-alpes.fr)](mailto:sophie.rousseaux@univ-grenoble-alpes.fr)1),

Emie Seyve ([emie.seyve@univ-grenoble-alpes.fr)](mailto:emie.seyve@univ-grenoble-alpes.fr)1),

Florent Chuffart ([florent.chuffart@univ-grenoble-alpes.fr)](mailto:florent.chuffart@univ-grenoble-alpes.fr)1),

Ekaterina Bourova-Flin ([ekaterina.flin@univ-grenoble-alpes.fr)](mailto:ekaterina.flin@univ-grenoble-alpes.fr)1),

Meriem Benmerad ([mbenmerad@chu-grenoble.fr)](mailto:mbenmerad@chu-grenoble.fr)1),

Marie-Aline Charles ([marie-aline.charles@inserm.fr)](mailto:marie-aline.charles@inserm.fr)2),

Anne Forhan ([anne.forhan@inserm.fr)](mailto:anne.forhan@inserm.fr)2),

Barbara Heude ([barbara.heude@inserm.fr)](mailto:barbara.heude@inserm.fr)2),

Valérie Siroux ([valerie.siroux@univ-grenoble-alpes.fr)](mailto:valerie.siroux@univ-grenoble-alpes.fr)1),

Remy Slama ([remy.slama@inserm.fr](mailto:remy.slama@inserm.fr)),

Jorg Tost ([tost@cng.fr)](mailto:tost@cng.fr)3),

Daniel Vaiman ([daniel.vaiman@inserm.fr)](mailto:daniel.vaiman@inserm.fr)4),

Saadi Khochbin ([saadi.khochbin@univ-grenoble-alpes.fr)](mailto:saadi.khochbin@univ-grenoble-alpes.fr)1),

Johanna Lepeule ([johanna.lepeule@univ-grenoble-alpes.fr)^*^](mailto:johanna.lepeule@univ-grenoble-alpes.fr)*)

and the EDEN mother-child cohort study group.

**Figure S1, Figure S2, Figure S3, Figure S4, Figure S5**

**Table S1, Table S2, Table S3, Table S4**

**Supplementary Figure S1: cellular heterogeneity estimated by the RefFreeEWAS method on 668 placenta samples from the EDEN cohort (A) contribution (%) of each latent variable (B) Pearson correlation between the 6 latent variables.**

**
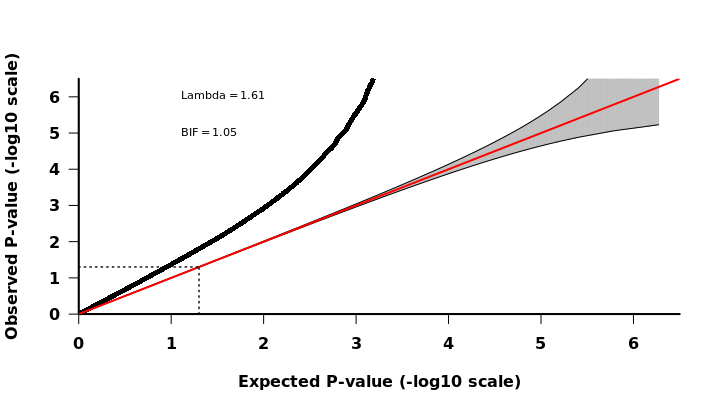
**

**Supplementary Figure S2: Q-Q plot, genomic inflation factor (lambda) and Bayesian inflation factor (BIF) for the association between each methylation site of the EWAS (425,878 CpGs) and tobacco smoking during pregnancy (current, never, former smoker).** Results were adjusted for child sex, parity, education level, season of conception, study center, maternal body mass index before pregnancy, maternal age at delivery, gestational duration, paternal smoking status at conception, batch, plate and chip and estimated cellular heterogeneity.

**Supplementary Figure S3**: **H3K4me3 (A), H3K4me1 (B) and H3K27ac (C) ChIPSeq signal of 3 placenta replicates.**

The top panels represent the mean signal values centered on our 203 DMRs +/-2kb (blue) and the mean signal values centered on the 420 random regions +/-2kb (green). The center panels represent heatmaps of the corresponding ChIP-Seq signals centered on our 203 DMRs +/-2kb (upper heatmaps) or on the 420 random regions+/-2kb (lower heatmaps).

**
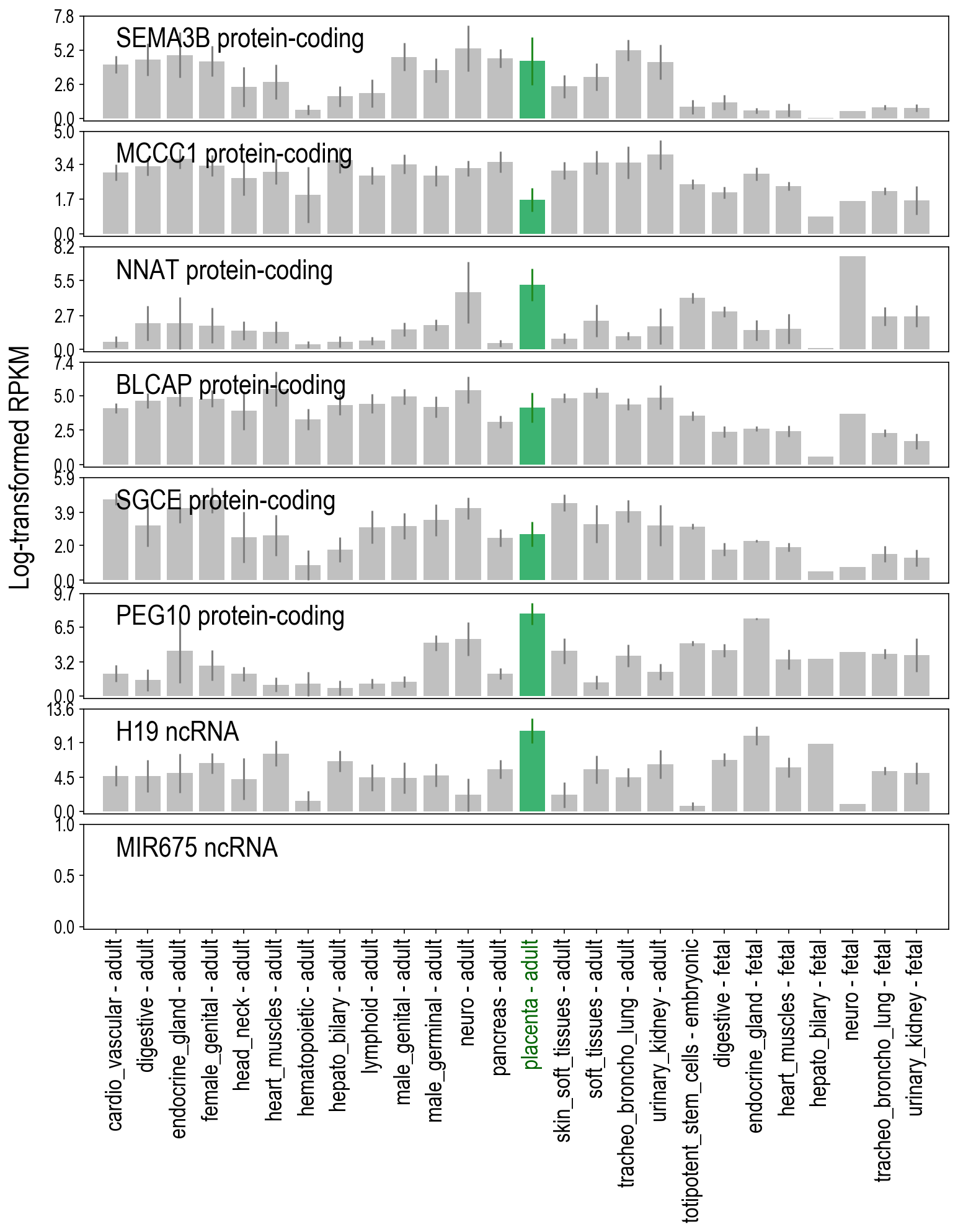
**


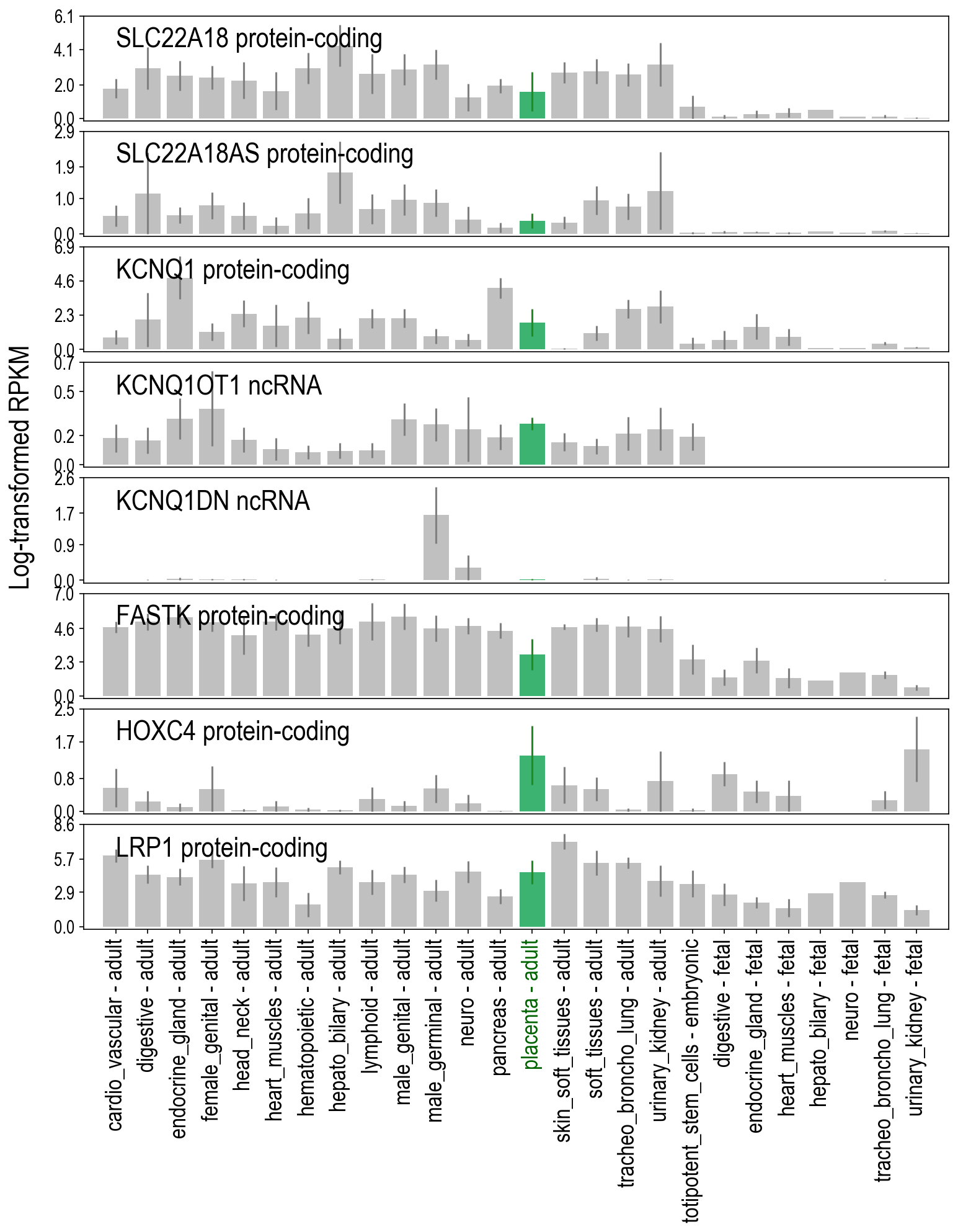


**Supplementary Figure S4 –** **Expression of genes in normal human tissues from RNA-seq data**

The barplots show average expression levels of the 16 imprinted genes overlapping our DMRs in normal tissues and development stages (adult, embryonic and fetal). For the sake of clarity, the plots are separated in two panels of 8 genes. Vertical lines on the top of the barplots represent standard deviations. Barplots representing expressions in placenta are in green. RNA-seq data in normal tissues were provided by GTEx portal and NCBI Sequence Read Archive (datasets PRJNA280600, PRJEB4337, PRJEB2445, PRJNA270632, GSE70741, GSE53096). The expression levels are represented in log-transformed RPKM (Read per Kilobase Million) values after addition of a pseudo count of 1, i.e. log-transformed RPKM = log2(1+RPKM). MIR675 has no expression in any of the analyzed samples.

**
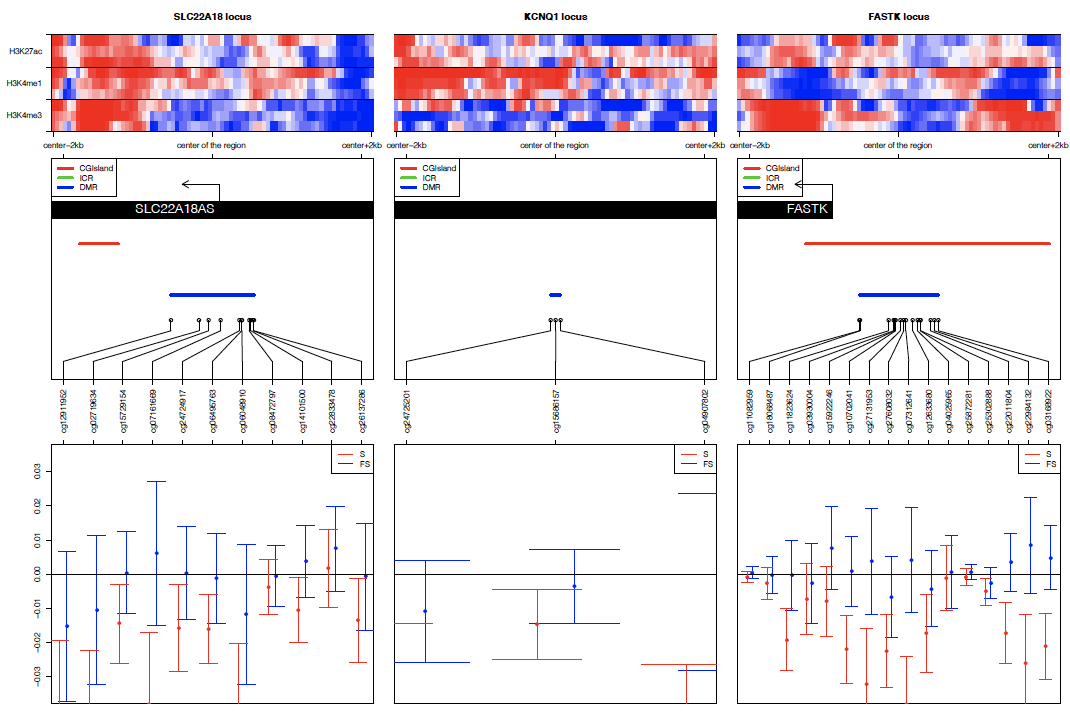

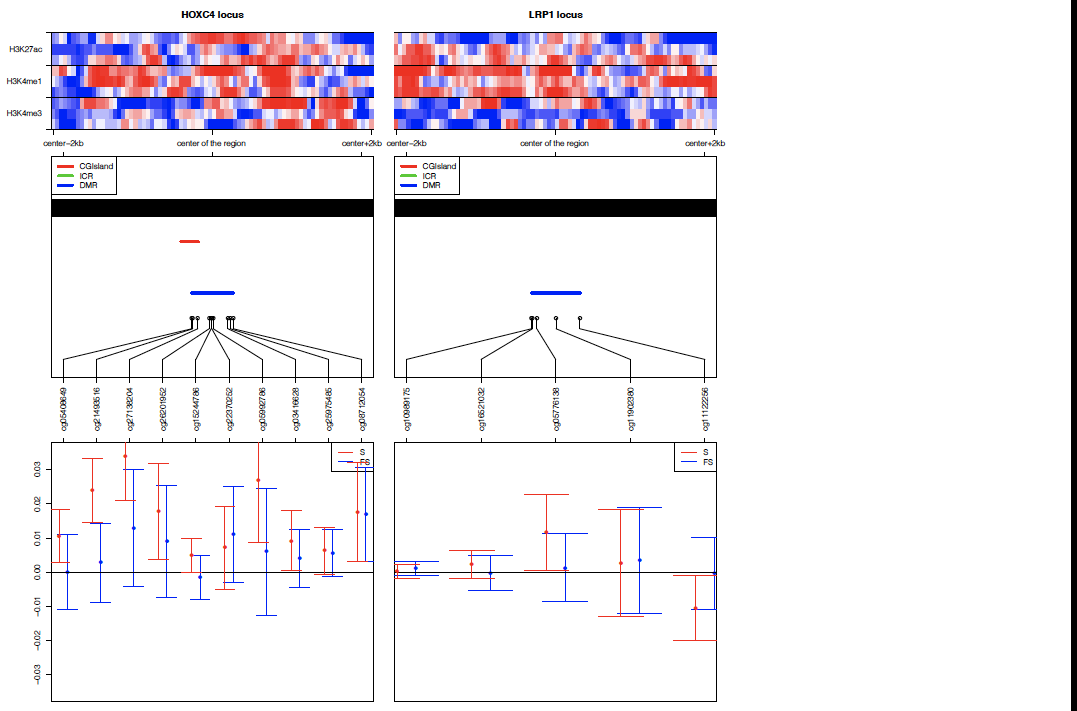
**

**Figure S5: The methylation levels of 7 imprinted loci are consistently modified following exposure to cigarette smoking.**

The left, center and right panels are respectively centered on the DMRs associated with the *NNAT* locus +/- 2kb, the *SGCE/PEG10* locus +/- 2kb and the *H19/MIR675* locus +/- 2kb.

The top panels show heatmaps respectively corresponding to H3K4me3, H3K4me1 and H3k27ac enrichments in placenta around the center of the region of interest +/- 2 kilobases. For each mark we downloaded triplicates from ENCODE data (see methods). We computed the enrichment matrices from bigWig files using the deepTools software and displayed it using a custom R script.

The middle panels show genes and CpG islands from the Illumina Infinium Human Methylation 450K BeadChip annotations, ICR from Pervjakova et al. *(70)*, the DMR we identified and the corresponding Illumina probes of the region of interest.

The lower panels show the methylation changes in smokers (S) (resp. Former Smokers (FS)) in red (resp. blue) compared to nonsmokers. The points represent the beta of the linear model and the error bars correspond to 2 standard deviations. The Y-axis represents the distributions of the variations of the regression coefficient (i.e. the associated change in DNA methylation level between 0 and 1) for each CpG within the DMRs selected by our analysis.

< Supplementary tables S1 to S4 are too large for being included in the manuscript. Please see excel file named Additional file 1. >

**Table S1: Metadata of analyzed ENCODE files.**

**Table S2: Results from the Epigenome Wide Association Study (EWAS): 1,800 CpGs differentially methylated between the three groups of women (nonsmokers, current smokers or former smokers) (p-value corrected for False Discovery Rate (FDR) <0.05)**

**Table S3: Results from the regional analysis using comb-p: 203 Differentially Methylated Regions including 1023 CpGs**

**Table S4: Comparison of the number of CpGs found significantly associated with smoking between the present EDEN study and previous studies conducted on placenta and cord blood**
